# Surgery

**Published:** 1892-05

**Authors:** 


SURGERY. ITS THEORY AND PRACTICE.—By William Johnson Walsham, F. R. C. S., Assistant Surgeon to St. Bartholomew's Hospital, etc. Third edition, revised and enlarged, with three hundred and eighteen illustrations. Philadelphia: P. Blakiston, Son & Co., 1891, pp. 15-748.

This excellent work is intended more for the student than the surgeon, but can be studied by both with benefit; it is a concise and efficient guide, and in addition is thoroughly practical.

It is abreast of the times in surgical pathology, and is ahead of most similar works in the theory of inflammation and the possibility of preventing it by the proper application of antiseptic precautions.

In the repair of fractures he describes the process of union as similar to that which occurs in the healing of a wound of the soft part by first intention, which is good sound teaching. The illustrations are well selected, and some of the original ones are very instructive, particularly the diagrams illustrating the collateral circulation.

All through the work special prominence has been given to those subjects with which every student and general practitioner who does any surgery, should be acquainted.

The book is well gotten up and the index is particularly good. W. F. W.

### A CASE OF CÆCAL HERNIA COMPLICATED BY INFLAMMATION OF THE VERMIFORM APPENDIX.

A case of this character is reported by Gangolphe (*Lyon Medical*, 24 Annee, tome lxix.) The hernia was one of gradual formation, and at the time it came under observation had descended into the scrotum of the right side. It was about the size of a child's head, and was acutely inflamed, although there were no signs of strangulation. An operation was performed, opening an abscess of the scrotum. On further search, the vermiform appendix was found, and was removed in spite of firm adhesions. The patient recovered.

In relation to hernia of the large intestine, the conclusions of Tuffier are of interest. He states that though congenital hernias of this nature may be without sac, peritoneal investment is found in all ruptures of slow formation. It is true that cases are reported where a sac has been absent, but none of these will stand careful study.

Gangolphe wishes, by his paper, to draw attention to the fact that appendicitis may occur in the sac of a hernia; that this will be indicated by inflammatory phenomena, together with suppuration, but without symptoms of strangulation; that operation is exceedingly difficult, on account of the firm adhesions, and that incision, if colotomy is determined upon, should generally be made upon the anterior inner surface of the tumor, since thus the danger of wounding the large intestine is lessened.—*Therapeutic Gazette*, March 15, 1892.

